# NIR-II light-activated two-photon squaric acid dye with Type I photodynamics for antitumor therapy

**DOI:** 10.1515/nanoph-2022-0482

**Published:** 2022-10-24

**Authors:** Kexin Wang, Yunjian Xu, Zhenjiang Chen, Huixian Li, Rui Hu, Junle Qu, Yuan Lu, Liwei Liu

**Affiliations:** Key Laboratory of Optoelectronic Devices and Systems of Guangdong Province & Ministry of Education, College of Physics and Optoelectronic Engineering Shenzhen University, Shenzhen, Guangdong Province, 518060, P. R. China; Department of Dermatology, Shenzhen Nanshan People’s Hospital and The 6th Affiliated Hospital of Shenzhen University Health Science Center, and Hua Zhong University of Science and Technology Union Shenzhen Hospital, Shenzhen, Guangdong Province, 518060, P. R. China

**Keywords:** hydroxyl radicals, hypoxia, squaric acid, Type I PDT

## Abstract

Photodynamic therapy (PDT) for hypoxic tumors has attracted wide attention owing to its noninvasiveness, easy maneuverability, and instantaneity. However, hypoxia in tumors and penetration depth of conventional ultraviolet light has greatly weakened its performance. To solve these problems, under NIR-II light irradiation, squaric acid nanoparticles (SQ NPs) with superior reactive oxygen, especially, hydroxyl radicals (•OH) production performance were first utilized for hypoxic tumor therapy. SQ NPs with intense light capture capability, intense NIR emission, and excellent photobleaching resistance show continuous •OH generation capabilities under NIR-II laser excitation. Through the superior PDT performance, the growth of hypoxic tumors was effectively inhibited, and the survival rate of mice was improved. This work highlights the application of NIR-II photoexcitation in deep tissue type I photodynamic therapy of hypoxic tumors, which will facilitate the development of hypoxic tumor PDT in deep depth.

## 1 Introduction

Tumor is a malignant disease that seriously threatens people’s health because of its high morbidity and mortality [1, 2]. However, the current clinical methods for the treatment of tumors have a certain degree of side effects and complications [3]. Therefore, there is an urgent need to research and to invent effective solutions for tumor treatment. The study of a noninvasive tumor treatment method such as photodynamic therapy (PDT) has attracted widespread attention due to its noninvasiveness, easy maneuverability, and instantaneity [4–6]. In PDT, photosensitizers (PSs) were sensitized under specific light irradiation to generate singlet oxygen (^1^O_2_) under oxygen (O_2_) conditions for type II PDT or free radical (such as hydroxyl radicals, •OH) for type I PDT [7, 8]. Type II PDT was widely studied because of the abundance of type II PSs [9, 10]. However, hypoxic conditions of tumor seriously weakened type II PDT performance [11–13]. Although type I PDT was independent of O_2_, few works on type I PSs were reported owing to the limitation of energy level matching and the deficiency of Type I PSs [14–16]. In addition, compared to traditional PSs [8], such as porphyrins excited by ultraviolet light, and some near-infrared-I (NIR-I) (650–900 nm) phototherapeutic agents [17–19], NIR-II light (1000–1700 nm) activated nanostructure-based PSs for tumor therapy showed great advantages [20–23], such as low cell damage, deep tissue penetration for superior tumor therapy [24–30]. Therefore, to explore type I PSs excited under NIR-II light exhibited promising potential for highly efficient PDT performance of hypoxic tumor.

**Scheme 1:**
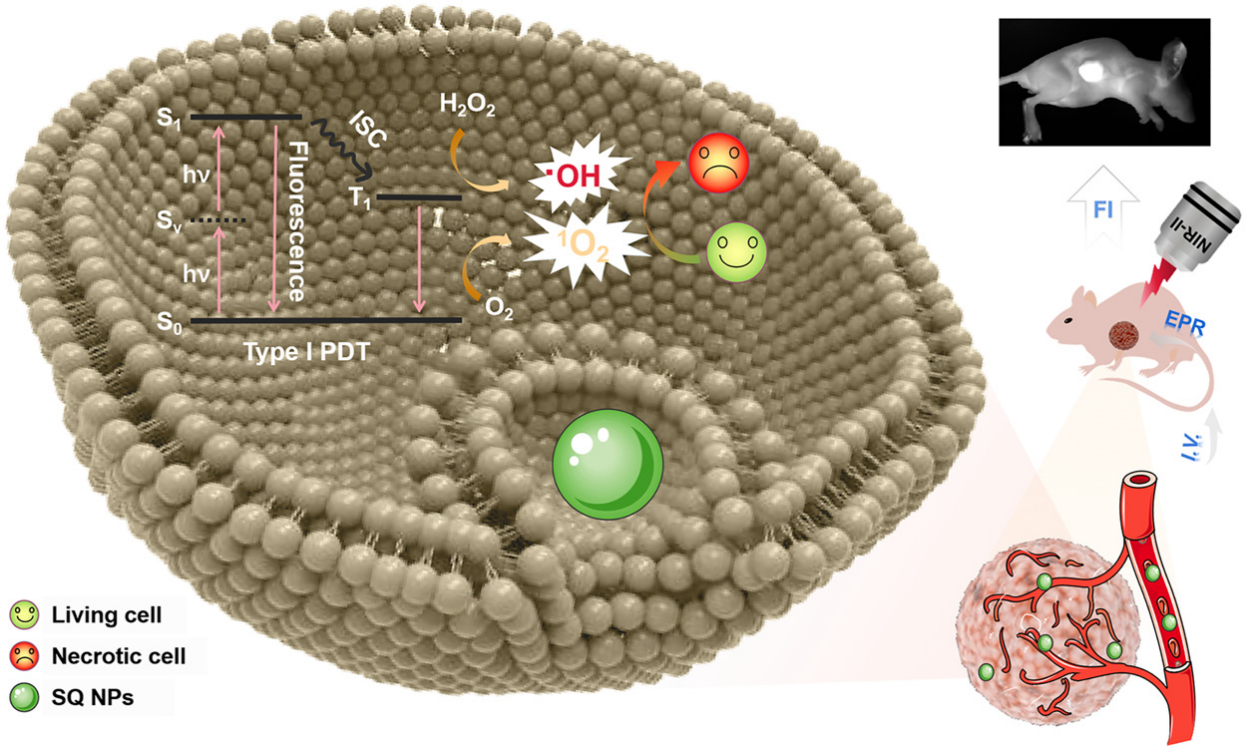
Schematic illustration of two-photon-activated type I PSs based on SQ dye for PDT of hypoxic tumor under irradiation of a NIR-II light.

Taking the above points into consideration, squaric acid (SQ) derivatives with dihydroxy cyclobutenedione skeleton were chosen as the candidate [31, 32]. They exhibited intense light capture capability, easily modified chemical structure, and controllable chemical and physical properties, and as a result, they have been widely applied in the fields of imaging, sensing, therapy, and photoelectric device [33–35]. Especially, SQ dye with donor-acceptor–donor structure usually exhibits a large two-photon absorption cross-section. In addition, pyridine-like salt framework usually exhibited excellent reactive oxygen species (ROS) (especially hydroxyl radical) generation performance [36–38], which endowed SQ derivatives promising potential as two-photon absorption type I PSs for PDT. Therefore, it is very important to explore SQ based type I PSs for PDT of hypoxic tumor under the irradiation of a NIR-II light.

In this work, under a NIR-II light irradiation, SQ nanoparticles (NPs) with excellent •OH generation capabilities were first found and used for type I PDT of the hypoxic tumors (Scheme 1). Compared to SQ dyes, its NPs showcased intense light capture capability and NIR emission, superior photobleaching resistance, and chemical stability, thus induces superior •OH generation capabilities under hypoxic conditions. With the above advantages, SQ NPs effectively inhibited the growth of tumor and improved the survival rate of mice under the irradiation of a NIR-II light with the guidance of fluorescence imaging. Besides, cytotoxicity analysis, routine analysis of blood, and H & E staining firmly confirmed the negligible toxicity of SQ NPs and superior PDT performance. These results demonstrated that SQ NPs could act as type I PSs for promising clinical applications.

## 2 Methods and experimental

### 2.1 Experimental information and synthesis of SQ dye

Phosphate buffered saline (HyClone, The United States), CCK-8 (Abbkine, The United States), SH-SY5Y, DMEM (Procell, China), fetal bovine serum (Gibco, The United States), trypsin-EDTA, penicillin-streptomycin (Mulgrave, Australia), ammonium molybdate, 9,10-anthracenediyl-bis(methylene)dimalonic acid (ABDA), 2,7-dichlorofluorescein diacetate (DCFH-DA), HKOH-1r (GlpBio, China), calcein-AM/PI double stain kit (Servicebio, China), LysoTracker^TM^ Green DND-26-Special Packaging (Thermo Fisher Scientific, The United States), ROS assay kit (Beyotime, China), Zetasizer-Nano-ZS90 (Malvern, United Kingdom), LAMBDA 750 UV/VIS/NIR Spectrophotometer (PerkinElmer, The United States), Fluorolog-3 Modular Spectrofluorometer (Horiba, Japan), Femtosecond pulsed laser (Coherent, The United States), CytoFLEX flow cytometer (Beckman, The United States), AnaeroPack-Anaero (Mitsubishi, Japan). All chemicals are analytical reagent grade and used without further purification.

Synthesis of 1-ethyl-2,3,3-trimethyl-3H-indolium bromide: 2,3,3-trimethyl-3H-indole (1.51 g, 9.60 mmol) and ethyl bromide (50.6 mmol) were dissolved in acetonitrile (20 mL) and the mixture was heated at 80 °C for 24 h. 1-ethyl-2,3,3-trimethyl-3H-indolium bromide was obtained by filtering and washing with cold acetonitrile. ^1^H NMR (500 MHz, DMSO-d6) δ 8.02–7.90 (m, 1H), 7.89–7.78 (m, 1H), 7.80–7.56 (m, 2H), 4.50 (q, *J* = 7.5 Hz, 2H), 2.83 (s, 3H), 1.54 (s, 6H), 1.45 (t, *J* = 7.0 Hz, 3H).

Synthesis of target square acid (SQ) dye: The mixture of squaric acid (0.57 g, 5 mmol) and triethyl orthoformate (10 mmol) in ethanol at 80 °C for 6 h. Then, 1-ethyl-2,3,3-trimethyl-3H-indolium bromide (10 mmol) was added. The obtained mixture then reacted under reflux for 12 h. The crude product was firstly purified by column chromatography (dichloromethane: methanol = 1: 0–40: 1). The final SQ dye was obtained by recrystallization in dichloromethane/cyclohexane. ^1^H NMR (500 MHz, DMSO-d6) δ 7.53 (d, *J* = 7.0 Hz, 1H), 7.40 (d, *J* = 7.0 Hz, 1H), 7.37–7.31 (m, 2H), 7.26 (t, *J* = 7.0 Hz, 1H), 7.11 (d, *J* = 7.5 Hz, 1H), 7.02 (t, *J* = 7.5 Hz, 1H), 5.79 (s, 1H), 5.52 (s, 1H), 4.13 (q, *J* = 13.5 Hz, 2H), 3.91 (q, *J* = 13.5 Hz, 2H), 1.68 (s, 6H), 1.54 (s, 6H), 1.28 (t, *J* = 7.0 Hz, 3H), 1.23 (t, *J* = 6.0 Hz, 3H).

### 2.2 Cell culture

Human bone marrow neuroblastoma cells (SH-SY5Y) were cultured in DMEM with 10% FBS and 1% streptomycin and penicillin at 37 °C and 5% CO_2_. The hypoxic conditions were created using AnaeroPack-Anaero. During the entire hypoxic simulation experiment, the cell plates or dishes were sealed into the AnaeroPack-Anaero bag containing O_2_ indicator and then placed in a cell culture incubator [39]. Before the experiment, SH-SY5Y cells (1 × 10^5^) were inoculated into a 20 mm diameter confocal culture dish for the next tests.

### 2.3 Cell viability assay

Cells were cultured in different concentrations of SQ NPs for 12 h, The CCK-8 kit was used to detect cell viability. All tests were conducted three times to obtain the average results.

### 2.4 ^1^O_2_ detection

The chemical probe (ABDA) was used to indirectly assess the singlet oxygen (^1^O_2_) generation capabilities of PSs by recording their absorption at 300–450 nm from the UV–Vis spectra [40]. In the experimental group, a mixture of PSs (27.5 μM) and ^1^O_2_ indicator (ABDA) was irradiated under 1150 nm laser (100 mW cm^−2^). ABDA solution without PSs was used as a control.

### 2.5 •OH detection

HPF was used as •OH indictor. The mixture of PSs (27.5 μM) and HPF (10 μM) into two same cuvettes were irradiated (1150 nm, 100 mW cm^−2^) under hypoxic and normoxic conditions, respectively [8]. And its maximal emission intensity was monitored with irradiation time.

### 2.6 Internalization and cell imaging of SQ NPs

SH-SY5Y cells were treated with SQ NPs (27.5 μM) for 15 min and their CLSM was obtained under a 561 nm light irradiation, which was used to evaluate the internalization of SQ NPs by SH-SY5Y cells. At the same time, the lysosomal indicator (LysoTracker^TM^ Green DND-26-Special Packaging) was used to co-stain the uptake of SQ NPs. CLSM was performed after 30 min incubation with the lysosomal indicator LysoTracker^TM^ Green in cells containing SQ NPs (27.5 μM). The excitation light was 488 nm, the fluorescence signal of LysoTracker^TM^ Green was collected in the range of 500–550 nm, and the fluorescence signal of SQ NPs was collected in the range of 570–620 nm. The co-localization of the two fluorophores in cells was analyzed by Image J. At the same time, the two-photon fluorescence imaging ability of SQ NPs was evaluated. SQ NPs were added to the cell, and the cell was imaged under two-photon microscopy with an increase of incubation time (5, 10, 15, 20, 25, 30 min). The excitation light was 1150 nm, and the two-photon fluorescence was collected through a 570–620 nm filter.

### 2.7 Cell viability assay under two-photon excitation

SH-SY5Y cells were treated with SQ NPs (27.5 μM) for 15 min, the selected area was scanned with 1150 nm f s light (100 mW cm^−2^) for 10 min. The cells were then cultured for 12 h, followed by adding calcein-AM and PI staining for 20 min, excited at 488 nm, the fluorescence signal of calcein-AM was collected in the range of 500–550 nm, and the fluorescence signal of PI was collected in the range of 570–620 nm. Because the fluorescence intensity of PI under this excitation light is much greater than that of SQ NPs, the fluorescence collected in the range of 570–620 nm is only the fluorescence intensity of PI by default.

### 2.8 *In vitro* ROS detection

Cells containing SQ NPs were placed under a two-photon femtosecond laser and irradiated with 100 mW cm^−2^ of 1150 nm light to induce ROS production. After irradiation, the ROS indicator (DCFH-DA) was added to the cells and incubated for 30 min for CLSM.

### 2.9 Flow cytometry

The cells with 80% confluency were trypsin-digested, centrifuged to remove the supernatant, and resuspended cells with the pre-chilled PBS buffer at 4 °C. The cells were centrifuged again to remove the supernatant and resuspended cells with binding buffer to achieve a concentration of 1 × 10^6^ cells mL^−1^. Then, 5 μL of Annexin V FITC and 2 μL of PI were added to 100 μL of cell suspension. After 15 min, the cells were detected at room temperature in the dark.

### 2.10 *In vivo* ^1^O_2_ detection

SQ NPs were intravenously injected into the mice, and 15 h later, 40 μmol kg^−1^ DCFH-DA was intratumorally injected, accompanied by irradiation (1150 nm, 100 mW cm^−2^, 10 min) after a further 15 min. The tumor was dissected, and the tumor was crushed. An appropriate amount of pure DMSO was added. After mixing evenly, the supernatant was collected by centrifugation, and its emission intensity at 528 nm was detected (*λ*_Ex_ = 485 nm).

### 2.11 PDT *in vivo*

Six Week-old female BALB/c nude mice were selected for the entire experiment. Nude mice were subcutaneously injected with SH-SY5Y cells (5 × 10^7^) to obtain the model of a subcutaneous tumor. All animal operations were performed under the guidance of the Medical Department of Shenzhen University. The mice were randomly divided into four groups for different experimental manipulations: (I) control group +75 μL PBS, (II) 75 μL PBS + irradiation (1150 nm, 100 mW cm^−2^, 10 min), (III) 75 μL SQ NPs, and (IV) 75 μL SQ NPs + irradiation (1150 nm, 100 mW cm^−2^, 10 min).

### 2.12 Histological analysis

The mice were sacrificed for histological analysis, and then main organs (heart, liver, spleen, lung, and kidney) and tumor were taken out and fixed in 4% paraformaldehyde solution. The fixed tissue was embedded in paraffin and then sectioned tissue was stained with hematoxylin and eosin, followed by observation with a CLSM. Blood samples were collected by enucleation 16 days after *in vivo* PDT for H & E and routine analysis of blood.

## 3 Results and discussion

### 3.1 Synthesis and photophysical property

**Figure 1:**
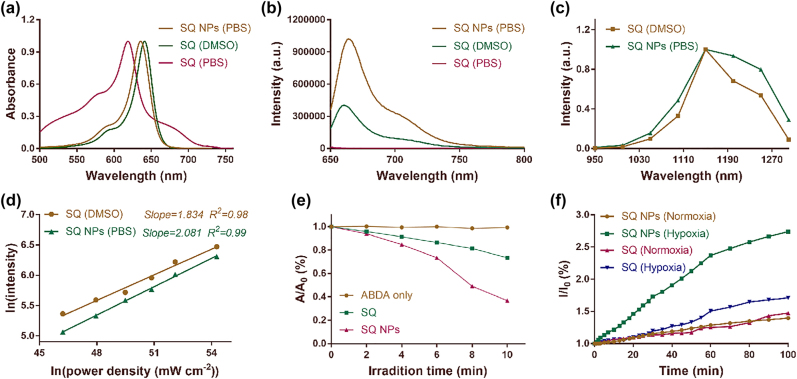
Photophysical properties of SQ dye. (a) Absorption spectra of SQ dye in DMSO and PBS, and SQ NPs in PBS. (b) Emission spectra of SQ dye in DMSO and PBS, and SQ NPs in PBS. Excitation: 635 nm. (c) Two-photon excitation spectra of SQ dye in DMSO and SQ NPs in PBS. (d) Laser-power-dependent luminescence of SQ dye and SQ NPs in PBS. Excitation: 1150 nm. (e) ^1^O_2_ generation capacity of SQ dye and SQ NPs in PBS. (f) •OH generating capacity of SQ dye and SQ NPs in PBS.

SQ dye was obtained in two steps according to referring to Scheme S1 previous report [41]. (I) The mixture of 2,3,3-trimethylindolenine and bromoethane in acetonitrile were used to prepare indole salt under reflux conditions. (II) The mixture of triacylglycerol, SQ skeleton, and indole salt in ethanol was used to obtain SQ dye under reflux conditions. To systematically explore the photophysical and photochemical property of SQ dye and its NPs, SQ and bovine serum albumin (BSA) with inherent biocompatibility and safety was used to prepare SQ NPs through electrostatic interaction and similarity compatibility principle. The concentration of SQ NPs was calculated as about 120 μM according to the standard curve for concentration-dependent absorption of SQ in dimethyl sulfoxide (DMSO) (Figure S1). TEM results displayed that SQ NPs were homogeneous spherical nanoparticles (Figure S2). The average hydrodynamic diameter of SQ NPs was measured as 72 nm by DLS (Figure S2), which indicated their promising capabilities of passive targeting tumors by enhanced permeability and retention (EPR) effect. The AFM measurement exhibits that the resulting SQ NPs have an average thickness of 3–7 nm and their typical lateral size is ≈75 nm (Figure S3), which is in a good agreement with the results obtained by dynamic light scattering (DLS) technique (Figure S2). SQ in DMSO showed strong light capture capability in the range of 525–675 nm with a maximal absorption peak (MAP) at 635 nm. The corresponding molar extinction coefficient (MEC) was 146,433 L cm^−1^ mol^−1^ (Figure 1a and Table S1). SQ dye in PBS exhibited poor absorbance and dispersity (Figure S4 and Table S1). For example, the MEC corresponding to its MAP (618 nm) was 41,441 L cm^−1^ mol^−1^, and many aggregates of free SQ are suspended in solution (Figures 1a and S4, and Table S1). By marked contrast, SQ NPs in PBS showcased excellent dispersity and strong light capture capability. Its corresponding MEC (641 nm) was 134,800 L cm^−1^ mol^−1^ (Figure 1a and Table S1). These results were similar to that of SQ in DMSO. In addition, SQ NPs in PBS showed much stronger emission than that of SQ in the range of 650–750 nm (Figure 1b). Besides, compared with SQ dye, SQ NPs also showed superior photobleaching resistance (Figure S5). These above advantages benefited from the NIR fluorescence imaging (FI) of SQ NPs *in vitro* and vivo.

SQ dyes with large two-photon absorption cross-sections were usually used for developing two-photon fluorescent probes. The two-photon absorption properties of SQ dye and its NPs were then explored. The excitation spectra of SQ dye and its NPs were drawn by a point-to-point way, respectively. As shown in Figure 1c, SQ dye and its NPs displayed a marked excitation peak in the range of 1100–1250 nm. Besides, SQ dyes and its NPs showed stronger emission when they were excited at 1150 nm compared to that at other excitation wavelengths, thus, which were chosen for the following tests. To confirm the two-photon absorption properties of SQ dye and it’s NPs at 1150 nm, the curve of their laser power-dependent emission was investigated referring to the previous report. Their slopes are 1.834 and 2.081 (Figure 1d), respectively, confirming their two-photon absorption properties.

### 3.2 Type I photodynamic under a NIR-II light irradiation

Indole salt skeleton endowed SQ dye and its NPs potential ROS production capabilities. Then, their ^1^O_2_ production performance was first explored by monitoring the changes in their characteristic absorption at 380 nm under irradiation of 1150 nm (100 mW cm^−2^) light. 9,10-anthracenediyl-bis(methylene)dimalonic acid (ABDA) acted as the singlet oxygen probe. Under the condition of light, the absorbance of ABDA shows a neglected change at 380 nm, showing that there is almost no ^1^O_2_ generation, and the characteristic absorption of ABDA and SQ dyes or SQ NP mixture shows a significant decline. The latter exhibited a more obvious downtrend, manifesting the excellent ^1^O_2_ production capabilities of SQ dye and SQ NPs (Figure 1e). Especially, the latter showcased better results, which was attributed to its intense light capture capability and superior photobleaching resistance (Figure S5 and Table S1). The ^1^O_2_ production capabilities of PSs were usually seriously limited owing to the hypoxic conditions of tumor. Therefore, the free radicals such as •OH generation performance, whose production was independent of O_2_, was a decisive factor for highly efficient PDT of the hypoxic tumor. Hydroxyphenyl fluorescein (HPF) was chosen as •OH probe because it can emit strong green fluorescence at 515 nm after reacting with •OH. Then, the •OH generation efficiency of SQ and its NPs were evaluated under hypoxic and normoxic conditions by recording the emission intensity of HPF at 515 nm under 1150 nm (100 mW cm^−2^) light. As shown in Figure 1f, the emission intensity at 515 nm of SQ dye or SQ NPs and HPF mixtures under normoxic conditions showed a poor increasing trend with irradiated time. After irradiation for 100 min, their emission intensity at 515 nm was 1.4 and 1.3 times of the starting value, respectively, indicating that SQ dye and SQ NPs can continuously generate •OH under normoxic conditions. By marked contrast, under hypoxic conditions, SQ dye or SQ NPs and HPF mixture showed more obviously improved emission intensity at 515 nm compared to that under normoxic conditions. For example, their emission intensity was 1.6 and 2.7 times of the starting number, respectively, further confirming that SQ dye and SQ NPs can continuously generate •OH. In addition, hypoxic conditions contributed to improving •OH generation performance of SQ dye and SQ NPs. Besides, SQ NPs showcased better •OH generation capabilities compared to that of SQ dye, which was attributed to its intense light capture capability, superior dispersity, and excellent photobleaching resistance. We further verified this result through electron spin resonance (ESR) measurements. The results obtained are as shown in Figure S6. SQ NPs hardly produce •OH without light irradiation. After 10 min of light irradiation (1150 nm), SQ NPs produce more •OH than that of SQ, indicating that SQ NPs have superior •OH generation capacity. Therefore, SQ NPs with superior ROS generation performance were chosen for the following tests.

### 3.3 Cellular internalization and cytotoxicity

**Figure 2:**
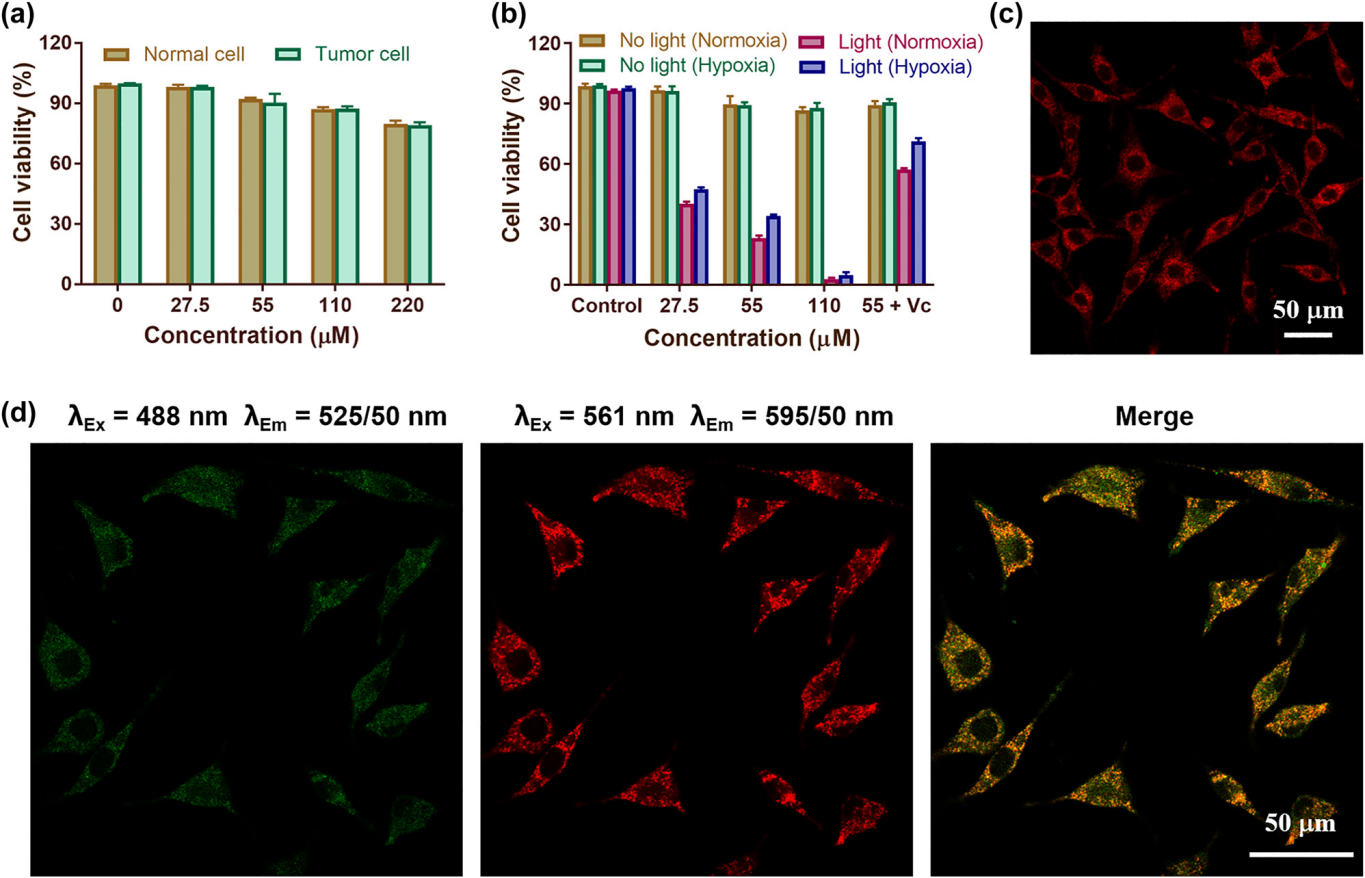
Cytotoxicity of SQ NPs on SH-SY5Y cells and their intracellular localization. (a) Dark toxicity of SQ NPs in normal cells (HEK29) and tumor cells (SH-SY5Y). The relative viabilities of SH-SY5Y cells incubated with different concentrations of SQ NPs for 12 h. (b) Phototoxicity of SQ NPs on SH-SY5Y cells with various treatments. (c) The intracellular imaging of SQ NPs. (d) Confocal laser scanning microscope of SHSY-5Y cells co-stained with SQ NPs and LysoTracker^TM^ Green.

In order to determine the dark cytotoxicity of SQ NPs to cells, different concentrations of SQ NPs were incubated with normal cells (HEK29) and tumor cells (SH-SY5Y) for CCK-8 assay. As shown in Figure 2a, the cell viability was higher than 89% even when the concentration of SQ NPs was as high as 55 μM, indicating that the dark toxicity of SQ NPs to HEK293 cells and SH-SY5Y cells is negligible. Phototoxicity tests of SQ NPs to SH-SY5Y cells were then carried out with the below treatments: (I) PBS, (II) SQ NPs (27.5 μM), (III) SQ NPs (55 μM), (IV) SQ NPs (110 μM), and (V) SQ NPs (55 μM) + Vc under normoxic and hypoxic conditions, respectively. Then, they were subjected to light irradiation or not. As shown in Figure 2b, in the absence of light irradiation, more than 89% of SH-SY5Y cells survived under both normoxic and hypoxic conditions, further confirming the negligible dark cytotoxicity of SQ NPs. Cell viability was greatly reduced under light irradiation compared to that without irradiation, indicating the superior PDT performance of SQ NPs. In addition, SQ NPs showed concentration-dependent phototoxicity to cells. For example, when the concentration of SQ NPs was 27.5 μM, the viabilities of SH-SY5Y cells were 40 and 47% under normoxic and hypoxic conditions, respectively, when the concentration of SQ NPs is 110 μM, the survival rates of SH-SY5Y cells under normoxic and hypoxic conditions are 3%, and 4%, respectively. The cellular phototoxicity increased with the increase of SQ NPs concentration. Besides, the presence of vitamin C (Vc) can promote the quenching of singlet oxygen, and the cell viability rebounded after the addition of Vc. For example, in normoxic conditions, the viability of SH-SY5Y cells was 23% after treatment with SQ NPs (55 μM) + light, and increased to 57% after adding Vc. Under hypoxic conditions, the cell viability was 34% after treatment with SQ NPs (55 μM) + light, and the cell viability reached up to 71% after the addition of Vc (Figure 2b), respectively. These results firmly confirm that SQ NPs with negligible cytotoxicity showed superior PDT effect *in vitro* even under hypoxic conditions.

To explore the intracellular distribution of SQ NPs, two-photon microscopy imaging was performed on SH-SY5Y cells after the addition of SQ NPs. As shown in Figures 2c, S7, and S8, with the increase of incubation time (5, 10, 15, 20, 25, 30 min), the intracellular fluorescence intensity increased, indicating that SQ NPs could enter the cells and its concentration in the cells gradually increased with time. To verify the specific distribution of SQ NPs in cells, the commercial lysosome indicator (LysoTracker^TM^ Green) was added to SH-SY5Y cells treated with SQ NPs for co-staining. As shown in Figure 2d, to a large extent, the green fluorescence produced by LysoTracker^TM^ Green matched well with the red fluorescence produced by SQ NPs (Pearsons correlation coefficient = 0.82). These results collectively indicated that SQ NPs could be taken by SH-SY5Y cells, and they are mainly distributed in lysosomes.

### 3.4 *In vitro* PDT

**Figure 3:**
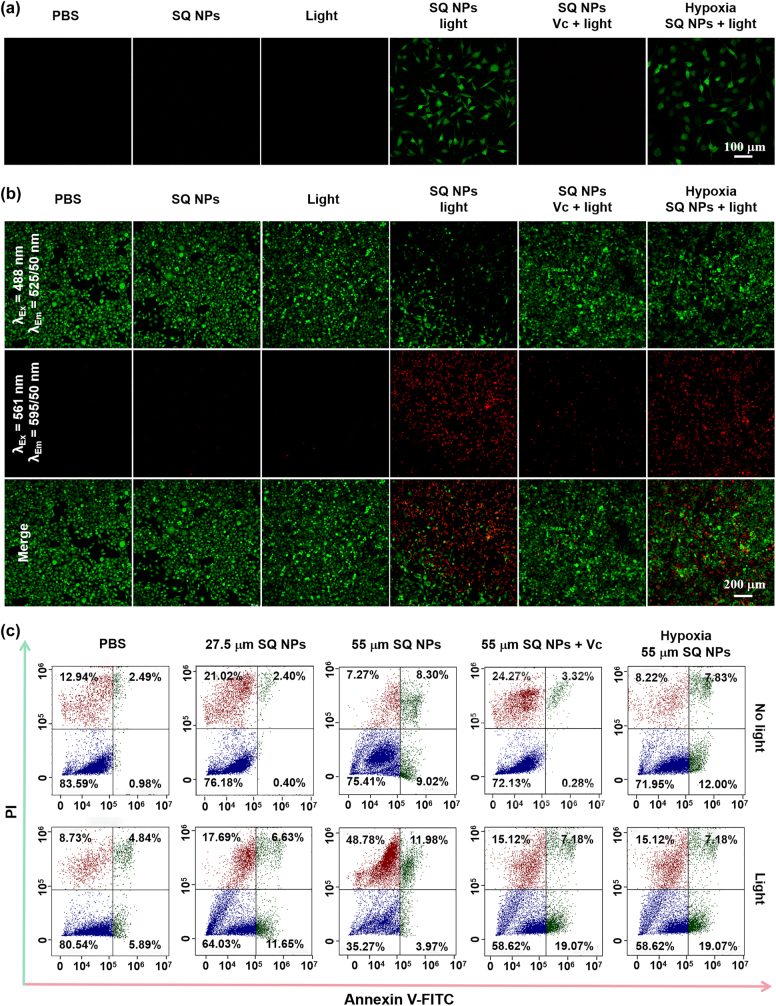
ROS production and PDT capacity of SQ NPs *in vitro* samples. (a) ROS production capacity of SQ NPs in SH-SY5Y cells treated with (I) PBS, (II) SQ NPs, (III) light, (IV) SQ NPs + light, (V) SQ NPs + Vc + light and (VI) hypoxia SQ NPs + light. (b) PDT capacity of SQ NPs on SH-SY5Y with treatments: (I) PBS, (II) SQ NPs, (III) light, (IV) SQ NPs + light, (V) SQ NPs + Vc + light and (VI) hypoxia SQ NPs + light. (c) Apoptosis analysis of SH-SY5Y cells with various treatments. (Green channel: 500–550 nm, excited at 488 nm; red channel: 570–620 nm, excited at 561 nm).

To further verify the potential PDT performance of SQ NPs in SH-SY5Y cells, ROS production performance of SQ NPs was first investigated. DCFH-DA with poor fluorescence was a ROS indicator, which could react with ROS and formed 2,7-dichlorofluorescein (DCF) with strong green emission, and the presence of Vc can quench ROS [42, 43]. In addition, HKOH-1r as an •OH indicator could rapidly and selectively target intracellular hydroxyl radicals [44]. When it reacts with •OH, it emitted green fluorescence. Therefore, ROS production capability and the presence of •OH under different treatments could be assessed by comparing their green fluorescence intensity in SH-SY5Y cells under a confocal laser scanning microscope (CLSM). SH-SY5Y cells incubated with DCFH-DA were randomly divided into five groups with the following treatments: (I) PBS, (II) SQ NPs, (III) Light, (IV) SQ NPs + Light, and (V) SQ NPs + Vc + Light, and (VI) hypoxia + SQ NPs + Light with HKOH-1r, respectively. As is shown in Figure 3a, no obvious green fluorescence was observed in groups (I), (II), and (III), indicating that ROS were not generated under these conditions. SH-SY5Y cells showed obvious green fluorescence in the group (IV) and (VI), but poor fluorescence in the group (V), indicating that SQ NPs exhibited excellent ROS generation ability in SH-SY5Y cells. Especially, the ROS included •OH, confirming the presence of promising Type I PDT.

Apoptosis is an important indicator of PDT performance [8, 45]. The PDT effect under hypoxic conditions was further determined by apoptosis experiments. SH-SY5Y cells cultured with calcein-AM and PI were divided into six groups with treatments: (I) PBS, (II) SQ NPs, (III) Light, (IV) SQ NPs + Light, (V) SQ NPs + Vc + Light, and (VI) hypoxia + SQ NPs + Light, respectively. Among them, the first three groups were served as control groups, and the (IV), (V), and (VI) groups were illuminated for 10 min. The experimental results showed that there was no obvious apoptosis of cells in control groups. (IV) and (VI) groups showed a large amount of apoptosis after light irradiation, and the number of apoptotic cells in (V) group was reduced after adding Vc, further indicating that SQ NPs had a good PDT effect (Figure 3b). The apoptosis rate of cells was further quantified by flow cytometry experiments. The results showed that in the absence of light, even when the probe concentration was higher than 55 μM, the cell viability was still at a high level of 75.41%. Under light irradiation conditions, the cell viability was 64.03 and 35.27%, corresponding to probe concentration as 27.5 and 55 μM. Cell viability increased to 58.62%, after the cell was treated with Vc + SQ NPs (55 μM) + irradiation (Figure 3c). These results are consistent with the cell imaging experimental results.

### 3.5 FI *in vivo*

**Figure 4:**
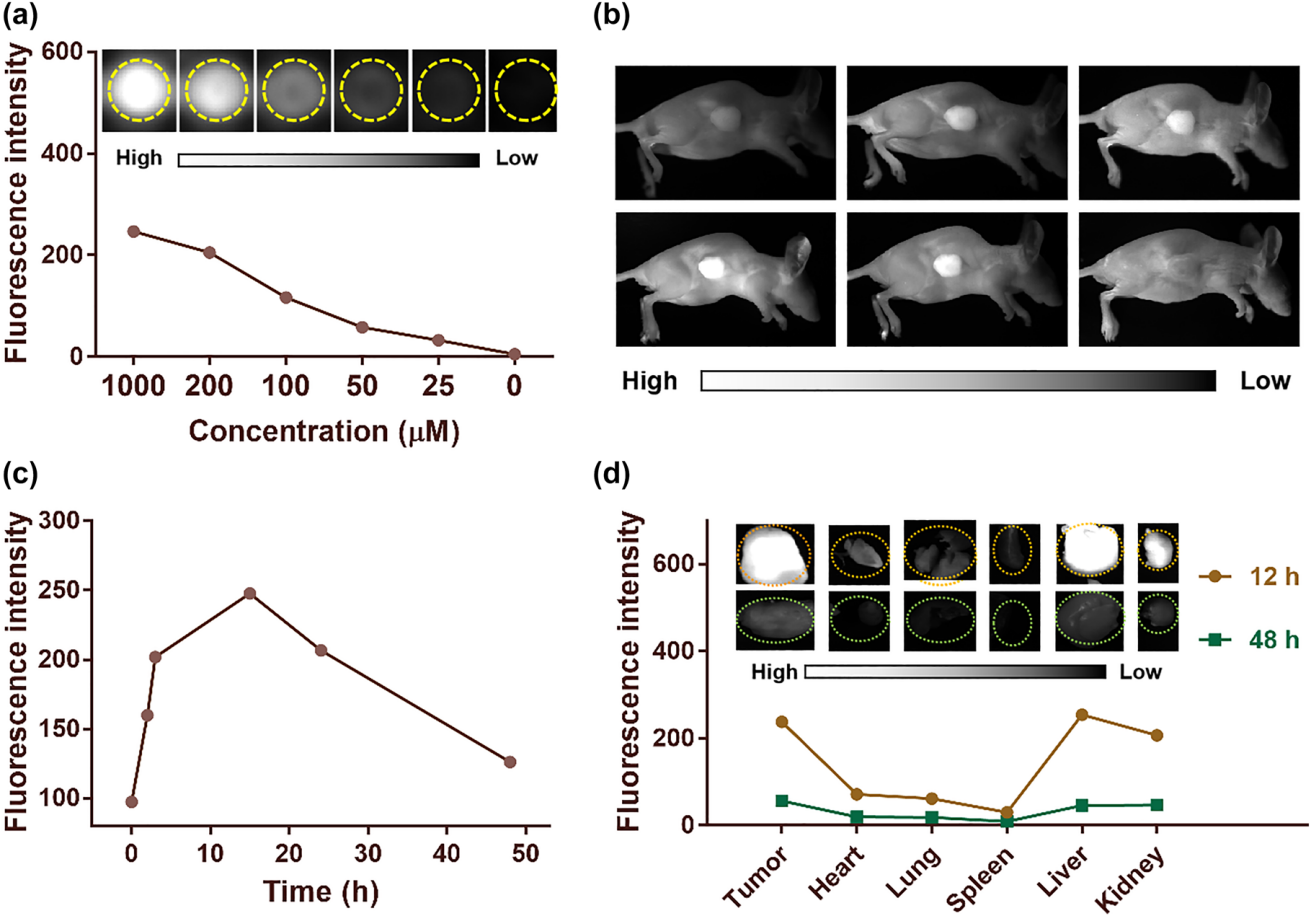
*In vivo* fluorescence imaging. (a) Fluorescence intensity of SQ NPs in different concentrations. (b) And (c) are fluorescence imaging images of mice and average fluorescence intensity of tumor sites after injection of SQ NPs with time. (d) Mean fluorescence intensities and (inset) images of mice tumor and major organs at 15 and 48 h after injection of SQ NPs.

SQ NPs showed a good PDT effect on SH-SY5Y cells *in vitro* under hypoxic conditions, which inspired us to further explore the PDT effect of SQ NPs on the tumor *in vivo*. In addition, relatively large-sized particles tend to aggregate in reticular tissues, such as the liver and kidney. Besides, SQ NPs showed concentration-dependent fluorescence signal (Figure 4a). Therefore, we first conducted the FI of mice to determining when the tumor should be irradiated after intravenous injection of SQ NPs before conducting *in vivo* PDT experiments. As displayed in Figure 4b and c, fluorescence signals intensity of tumor parts showed an increasing trend after intravenous injection of SQ NPs with time. It reached up to the brightest after injection for 15 h, which was 3 times of starting value, indicating that abundant SQ NPs were in tumor sites. Only weak or even negligible fluorescence signal intensity could be collected after injection of SQ NPs for 48 h, indicating that SQ NPs could be metabolized from the tumor. To explore the distribution of SQ NPs, the mice were killed after SQ NPs were intravenously injected for 15 and 48 h, respectively. The tumor and major organs such as heart, liver, spleen, lung, and kidney were obtained for FI (Figure 4d). There are strong fluorescence signals in tumor, liver, and kidney after intravenous injection of SQ NPs for 15 h, indicating that SQ NPs mainly accumulated in these organs and tumor. By marked contrast, poor fluorescence signals could be collected after the intravenous injection of SQ NPs for 48 h, indicating that SQ NPs could be metabolized from these organs and tumor.

### 3.6 *In vivo* PDT

**Figure 5:**
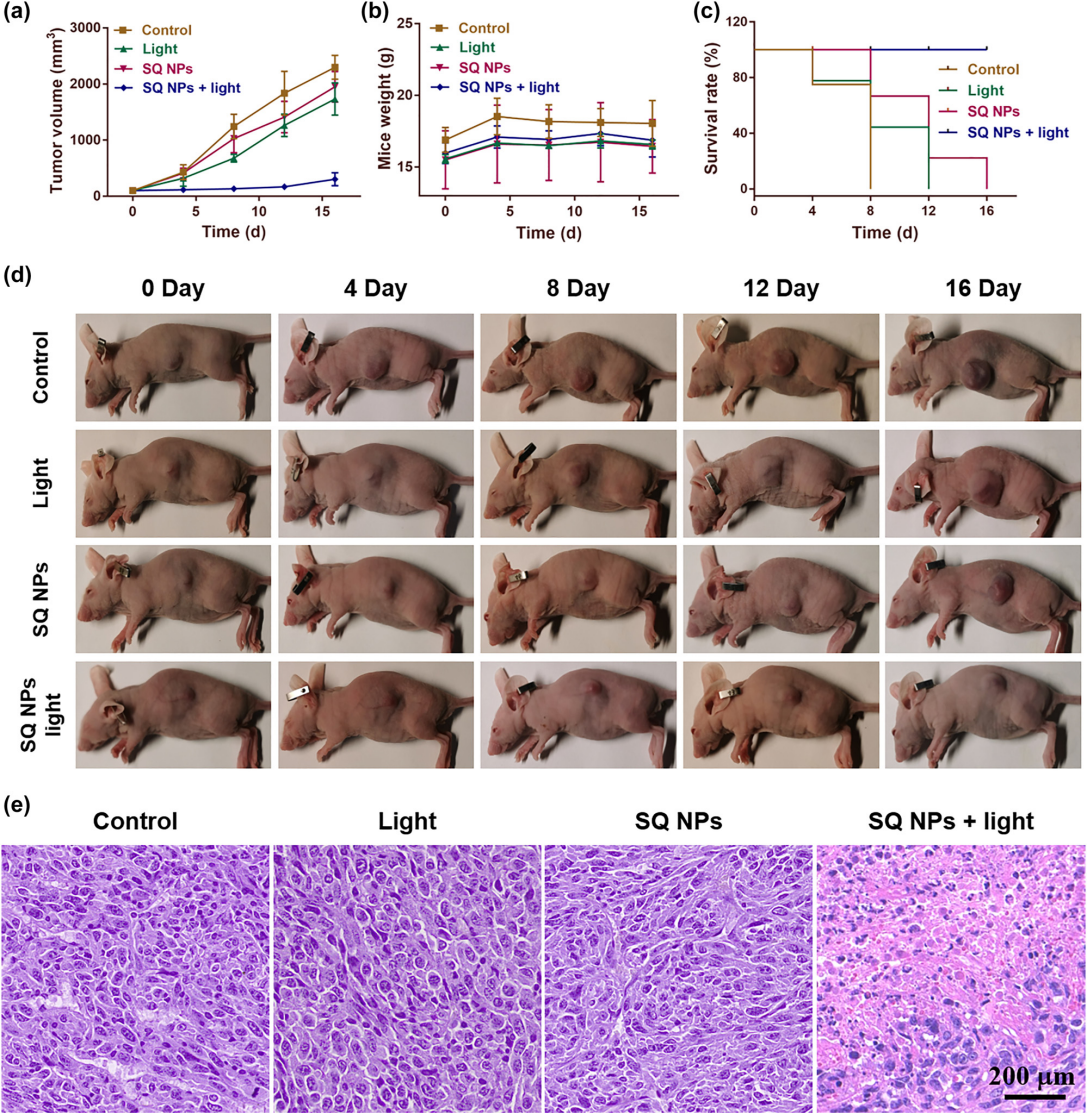
*In vivo* PDT. (a) Tumor volume. (b) Mice weight. (c) Mice survival. (d) Tumor size. (e) Tumor section staining of mice with various treatments. The scale is 200 μm.

Inspired by the superior PDT and EPR effect of SQ NPs, PDT of hypoxic tumor were then carried out. We first established subcutaneous tumor models by subcutaneous injection of SH-SY5Y cells in BALB/c mice. The *in vivo* fluorescence imaging showed that SQ NPs could effectively accumulate in the tumor site after SQ NPs was intravenously injected into mice for 15 h. Then, the antitumor efficacy of SQ NPs against SH-SY5Y tumor-bearing immunocompetent BALB/c mice *in vivo* was investigated. The mice were randomly divided into four groups: (I) PBS, (II) light, (III) SQ NPs, and (IV) SQ NPs + light. Mice body weight, tumor volume, and mice state are important elements to value PDT performance, which were recorded every four days. During PDT, the tumor of mice in groups I–III continued to grow rapidly, and their size was approximately 10 times of the starting value, indicating that light or SQ NPs had no significant effect on tumor volume changes. Compared with groups I–III, the mice tumor volume of group IV increased slowly, indicating that SQ NPs could effectively inhibit tumor growth under irradiation (Figures 5a and S9). Additionally, the mice body weight was monitored during the PDT process and similar weight changes were found for all groups pointing toward low systemic toxicity of SQ NPs (Figure 5b) In addition, the survival rate of mice in groups I, II, and III began to decline after 4 days of treatment, and none of the mice survived while 100% of mice survived in group IV after final PDT (Figure 5c). All mice showed excellent status, further confirming that SQ NPs and light had negligible dark toxicity in mice.

### 3.7 H & E staining and routine analysis of blood

To further evaluate the PDT effect of SQ NPs on the tumor and the promising toxicity of SQ NPs and various treatments, one mouse of each group were used for collecting tumor and major organs for H & E staining (Figures S10 and 5e) after all mice were sacrificed. And three mice from each group were used for collecting blood for routine analysis of blood. The H & E staining results of all major organs and tumor in groups I–III showed that no obvious apoptotic and necrotic tumor cells could be observed, further confirming negligible dark toxicity of SQ NPs and various treatments. In marked contrast, many of the apoptotic and necrotic cells, and obvious intercellular spaces could be seen in the tumor slices of group IV (Figure 5e), further demonstrating the superior PDT effect of SQ NPs on mice tumor. Besides, the key parameters including white blood cells (WBC), lymphocytes (Lymph), monocytes (Mon), neutrophils (Gran), etc. of blood showed no significant statistical distribution differences between each group (Figure S11), further confirming the negligible dark toxicity of SQ NPs and various treatments. The above study confirmed that the SQ NPs with reliable biosafety showed promising PDT effects on the hypoxic tumor, thus demonstrating their great potential in biological applications.

## 4 Conclusions

In this work, SQ NPs was firstly used as NIR-II light-activated (1150 nm) two-photon PSs for Type I PDT of the hypoxic tumor. The *in vitro* and *in vivo* results demonstrated that SQ NPs could efficiently inhibit tumor growth. This work gives new insight into the designing novel PSs to bypass the limitation of O_2_ dependent and light penetration depth issues for efficient tumor therapy. *In vivo* studies revealed that SQ NPs exhibited irreversible cytotoxicity against tumor tissue in NIR-II light excited two-photon PDT, resulting in ablation of apparent solid tumor. These results indicate that the designed and developed high-efficiency PSs can be used as novel PDT PSs with potential application value in future clinical treatment.

## Supplementary Material

Supplementary Material Details
