# Erratum to: NIR-II light-activated two-photon squaric acid dye with type I photodynamics for antitumor therapy

**DOI:** 10.1515/nanoph-2022-0669

**Published:** 2022-11-09

**Authors:** Kexin Wang, Yunjian Xu, Zhenjiang Chen, Huixian Li, Rui Hu, Junle Qu, Yuan Lu, Liwei Liu

**Affiliations:** Key Laboratory of Optoelectronic Devices and Systems of Guangdong Province & Ministry of Education, College of Physics and Optoelectronic Engineering Shenzhen University, Shenzhen, Guangdong Province, 518060, P. R. China; Department of Dermatology, Shenzhen Nanshan People’s Hospital and the 6th Affiliated Hospital of Shenzhen University Health Science Center, and Hua Zhong University of Science and Technology Union Shenzhen Hospital, Shenzhen, Guangdong Province, 518060, P. R. China

After the publication of this paper [1], the author found a diagram placement error in Figure 3c, which has been corrected here.

**Figure 3:**
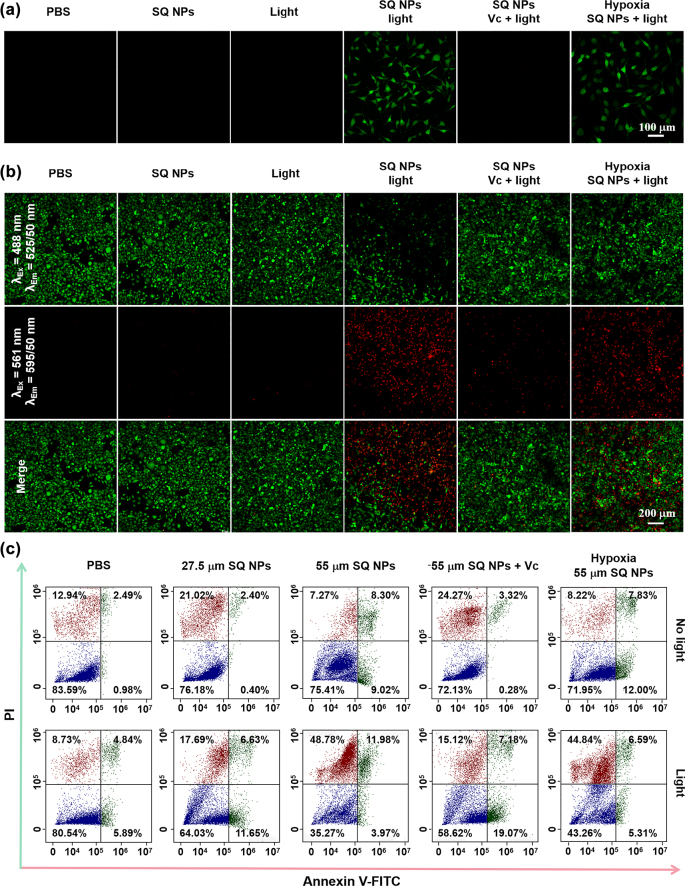
ROS production and PDT capacity of SQ NPs *in vitro* samples. (a) ROS production capacity of SQ NPs in SH-SY5Y cells treated with (I) PBS, (II) SQ NPs, (III) Light, (IV) SQ NPs + Light, (V) SQ NPs + Vc + Light and (VI) hypoxia SQ NPs + Light. (b) PDT capacity of SQ NPs on SH-SY5Y with treatments: (I) PBS, (II) SQ NPs, (III) Light, (IV) SQ NPs + Light, (V) SQ NPs + Vc + Light and (VI) hypoxia SQ NPs + Light. (c) Apoptosis analysis of SH-SY5Y cells with various treatments. (Green channel: 500–550 nm, excited at 488 nm; red channel: 570–620 nm, excited at 561 nm).
