# Supplementary material for: NIR-II light-activated two-photon squaric acid dye with Type I photodynamics for antitumor therapy
Source: Nanophotonics. 2022 Oct 24;11(22):5089–100. doi: 10.1515/nanoph-2022-0482 (PMC11501322; doi:10.1515/nanoph-2022-0482)
Supplement: Supplementary file 1 — Supplementary Material Details [file j_nanoph-2022-0482_suppl.pdf]

Supporting Information

NIR-II Light-activated Two-photon Squaric Acid Dye with Type I Photodynamics for Antitumor Therapy

Kexin Wang[a,#], Yunjian Xu[a,#], Zhenjiang Chen[a], Huixian Li[a], Rui Hu[a], Junle Qu[a], Yuan Lu[b,] and Liwei Liu[a,*]*

[a]Key Laboratory of Optoelectronic Devices and Systems of Guangdong Province & Ministry of Education, College of Physics and Optoelectronic Engineering Shenzhen University, Shenzhen, Guangdong Province, 518060, P. R. China

[b]The Sixth People's Hospital of Shenzhen, Shenzhen 518052, China

[*]E-mail: liulw@szu.edu.cn; chfsums@163.com

[#]These authors contributed equally to this work.

Contents

Part I. Supplementary Tables and Figures

Part I. Supplementary Tables and Figures

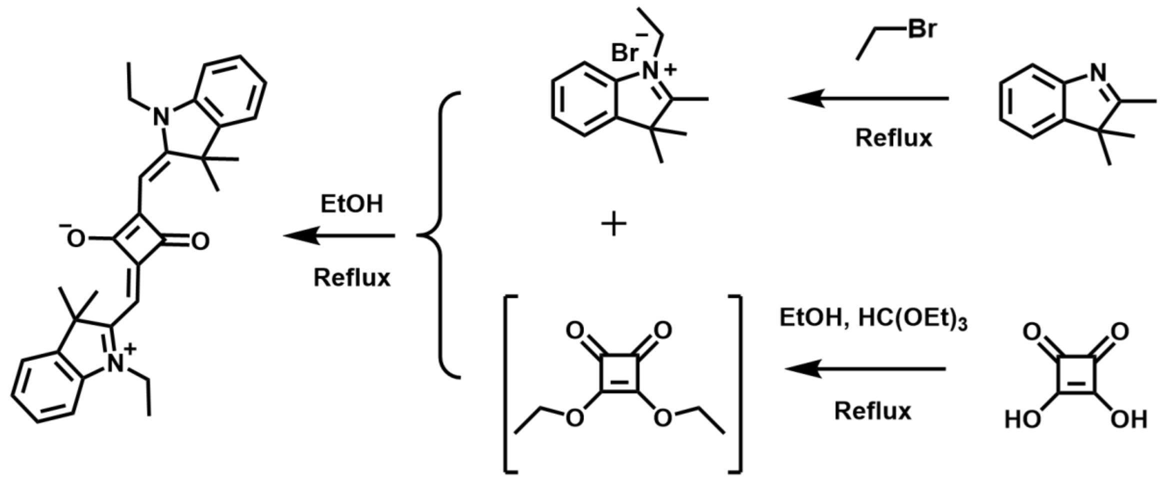

Scheme S1. The synthetic route to compound **SQ Dye**.

^{1}H NMR of SQ dye: (500 MHz, DMSO-d6) δ 7.53 (d, J = 7.0 Hz, 1H), 7.40 (d, J = 7.0 Hz, 1H), 7.37-7.31 (m, 2H), 7.26 (t, J = 7.0 Hz, 1H), 7.11 (d, J = 7.5 Hz, 1H), 7.02 (t, J = 7.5 Hz, 1H), 5.79 (s, 1H), 5.52 (s, 1H), 4.13 (q, J = 13.5 Hz, 2H), 3.91 (q, J = 13.5 Hz, 2H), 1.68 (s, 6H), 1.54 (s, 6H), 1.28 (t, J = 7.0 Hz, 3H), 1.23 (t, J = 6.0 Hz, 3H).

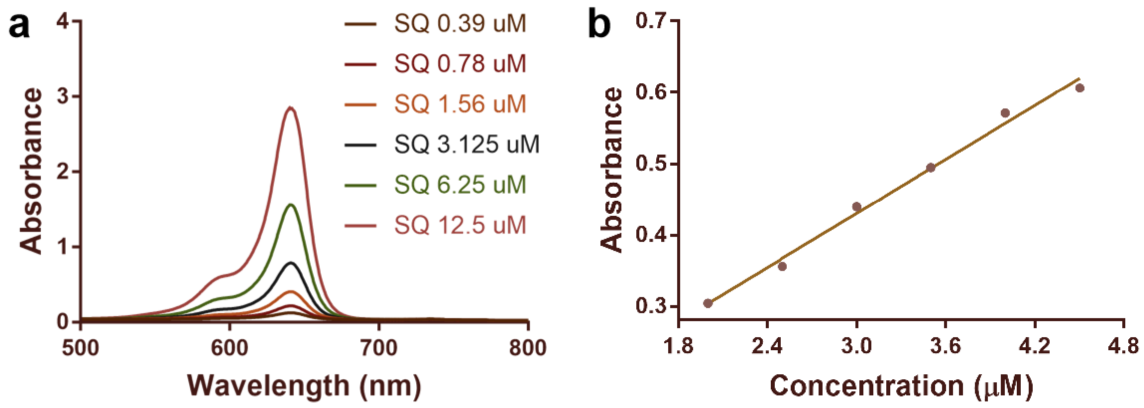

Fig. S1: (a) Absorption spectra of **SQ** in DMSO with different concentrations. (b) Concentrations-dependent maximal absorbance of **SQ** in DMSO (λ_{Ex} = 635 nm).

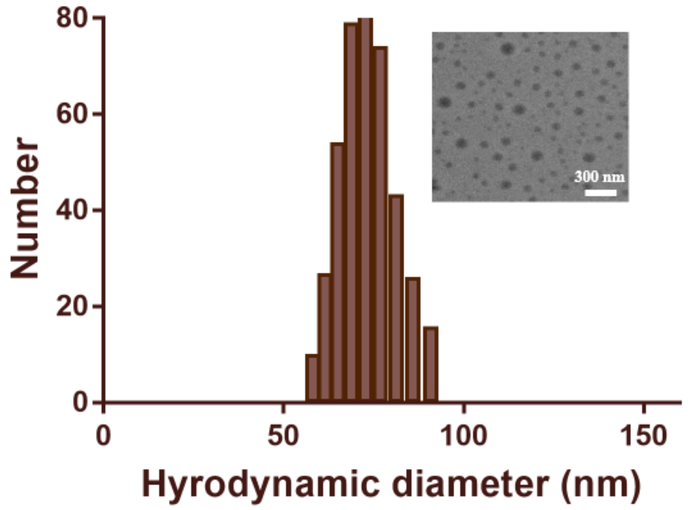

Fig. S2: DLS of **SQ NPs** in PBS (pH = 7.4). Inset was TEM images of **SQ NPs**.

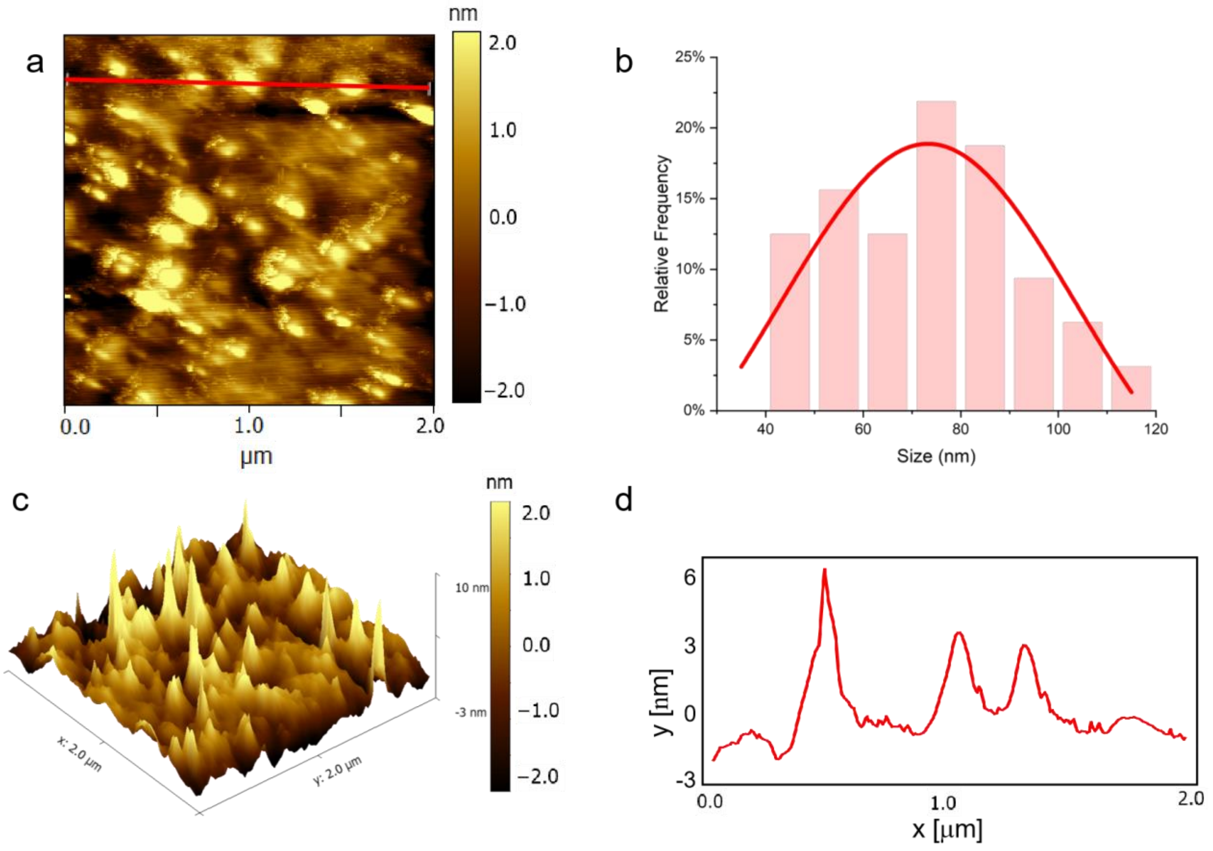

Fig. S3: Basic characterization of SQ NPs. (a) The AFM images. (b) Size distribution obtained from AFM images. (c) 3D image. (d) The corresponding height profiles along the red lines in (a).

Table S1: Photophysical and photochemical properties of **samples**.

Samples	Abs. (nm)	ε (L mol^{-1} cm)	E_m. (nm)
**SQ NPs (H_2O)**	635	134800	664
**SQ (DMSO)**	641	146433	660
**SQ (H_2O)**	618	41441	648

Table S2: Performance Comparison of **SQ NPs**, Other Nanomaterials and Photosensitizers.

Samples	Preparation steps	Penetration depth	Tumor inhibition rate	Ref.
**SQ NPs**	BSA+SQ $\xrightarrow{\text{electrostatic adsorption}}$ SQ NPs	1150 nm	85%	
**ZrC NSs**	ZrC $\xrightarrow{\text{probe sonicate + bath sonicate}}$ ZrC-NS<br>Niflumic acid+EDC·HCl+DMAP+SN38 $\xrightarrow{\text{stirring + drying}}$ SN38 Nif<br>2 PEG-NH_2+ZrC NSs $\xrightarrow{\text{electrostatic adsorption}}$ PEG-ZrC<br>ZrC-PEG-NH_2+ SN38 Nif $\xrightarrow{\text{stirring}}$ ZrC NSs	1064 nm	75%	[1]
**BPNSs**	BPPNs $\xrightarrow{\text{centrifugation + drying}}$ BPPNs<br>BPPNs+PEG-NH_2 $\xrightarrow{\text{centrifugation + resuspension}}$ BPNSs	808 nm	25%	[2]
**PDA@CP-PEG**	PDA $\xrightarrow{\text{soaking + drying}}$ PDA@CP<br>PDA@CP+PEG–NHS–PDA $\xrightarrow{\text{centrifugation + drying}}$ PDA@CP-PEG	808 nm	80%	[3]

The tumor inhibition rate is calculated by the follow equation:

tumor inhibition rate = (the tumor volume in treatment group/the tumor volume in control group)*100%.

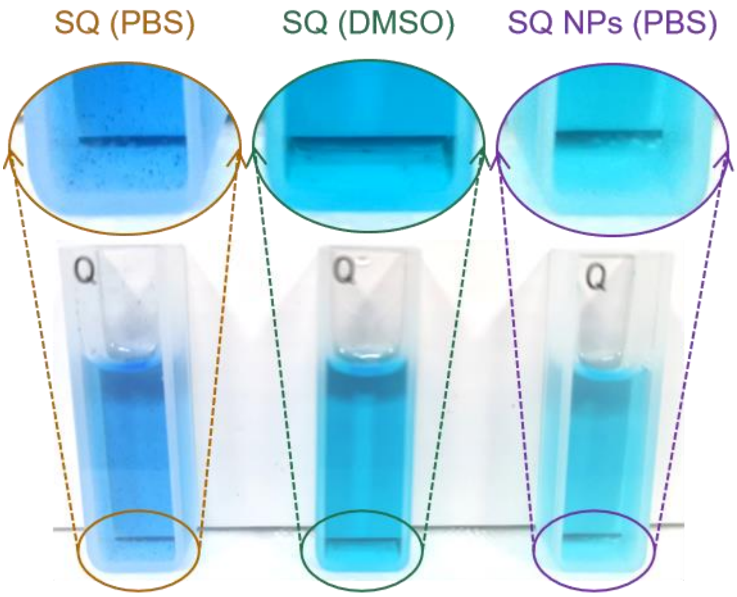

Fig. S4: Representative photographs of **SQ dye**. Inset is a zoomed-in photograph of the portion in the cuvette.

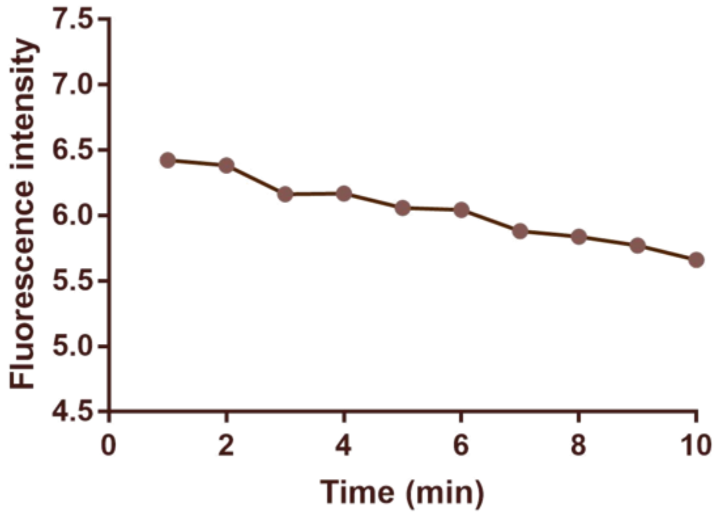

Fig. S5: Intracellular photobleaching resistance of **SQ NPs** with irradiation (100 mW cm^{-2}, 1150 nm) in different times, respectively.

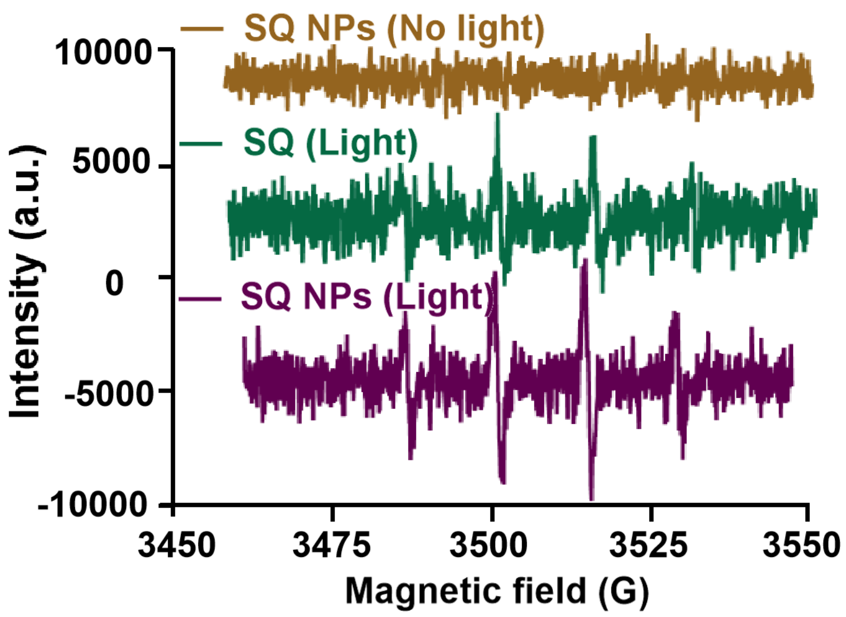

Fig. S6: DMPO spin-trapping ESR spectra recorded with SQ and SQ NPs in H2O dispersion (for DMPO-•OH) under hypoxic conditions.

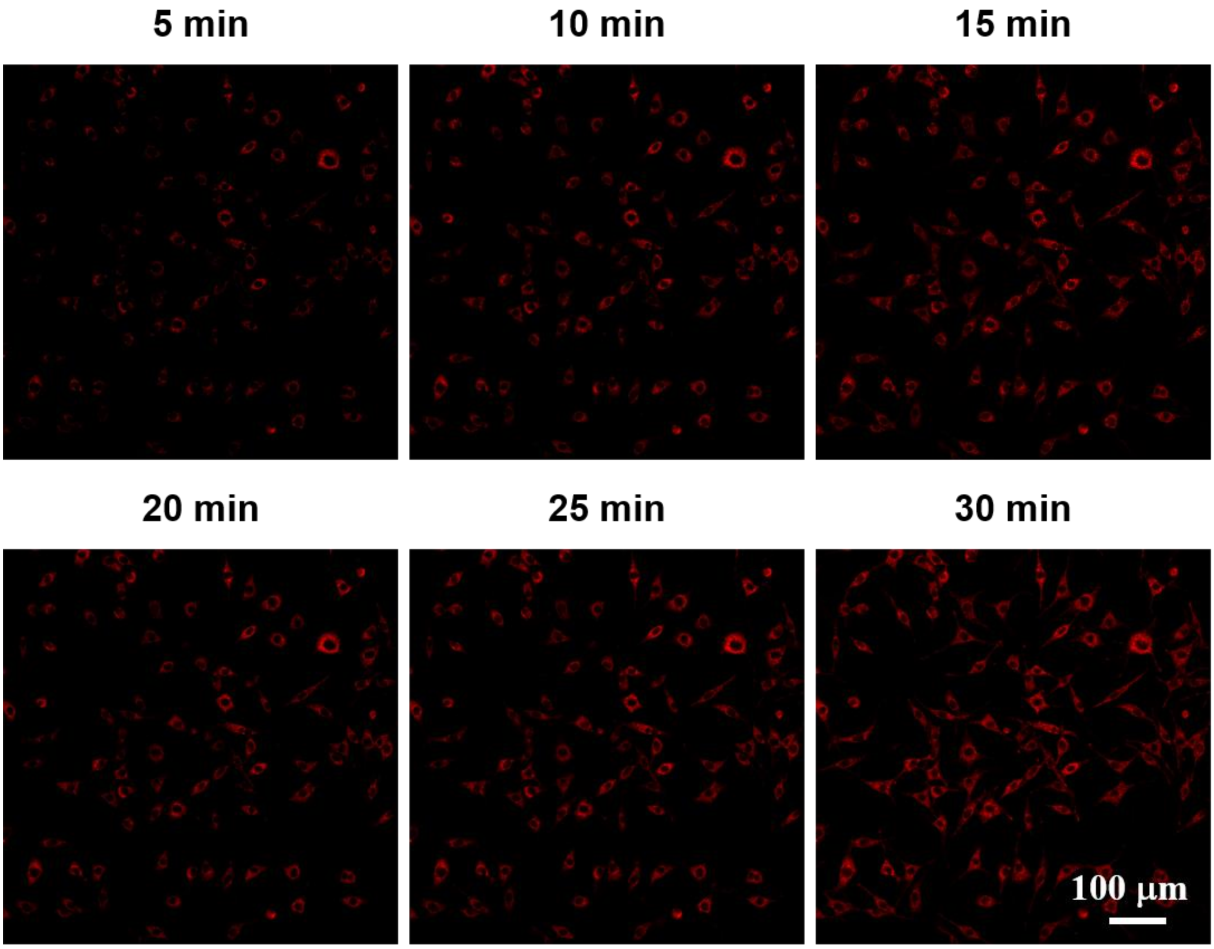

Fig. S7: Cellular uptake of **SQ NPs**. SH-SY5Y cells were incubated with **SQ NPs** at different times under normoxic conditions, respectively.

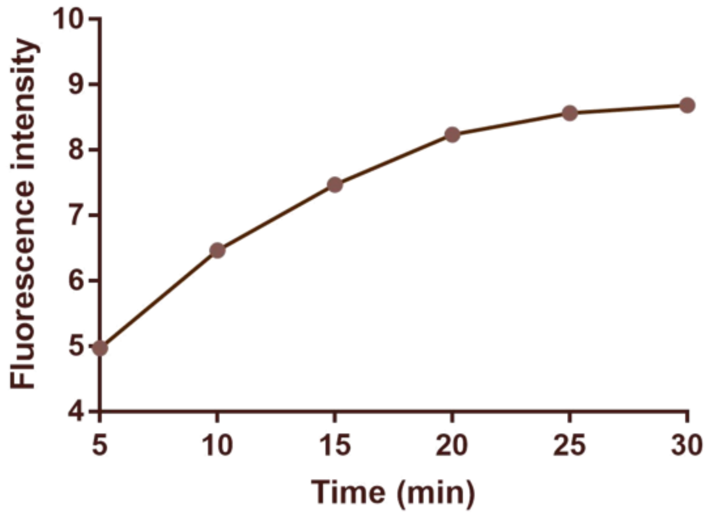

Fig. S8: The intracellular fluorescence intensity of **SQ NPs**.

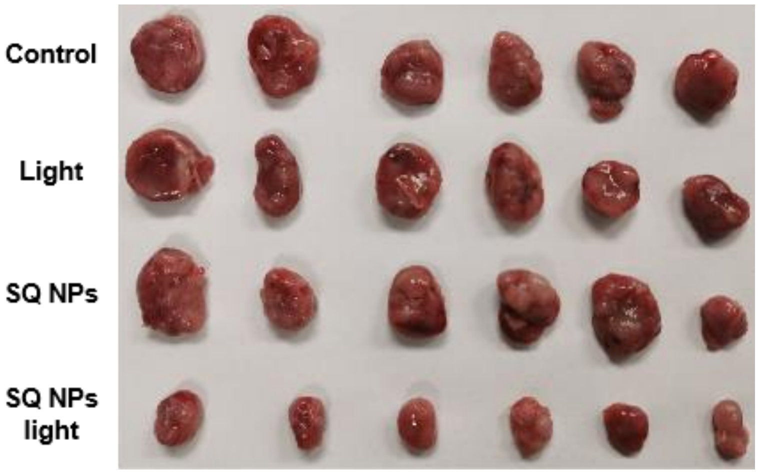

Fig. S9: Photographic image of tumors harvested from mice at 16 days after different treatments.

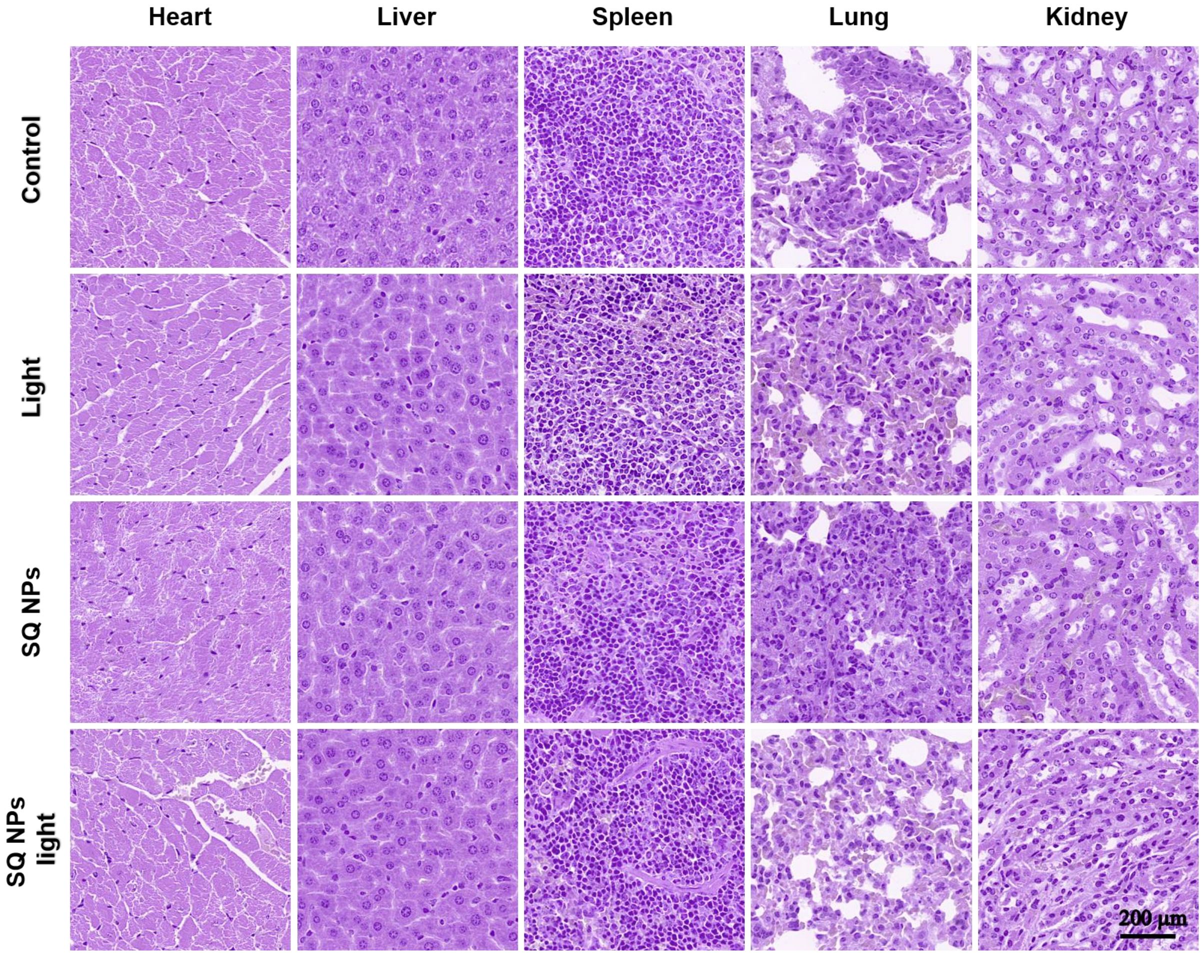

Fig. S10: Histological analysis of hematoxylin-eosin staining of heart, liver, spleen, lung, and kidney tissue obtained from tumor-bearing mice in each group. The scale is 200 μm.

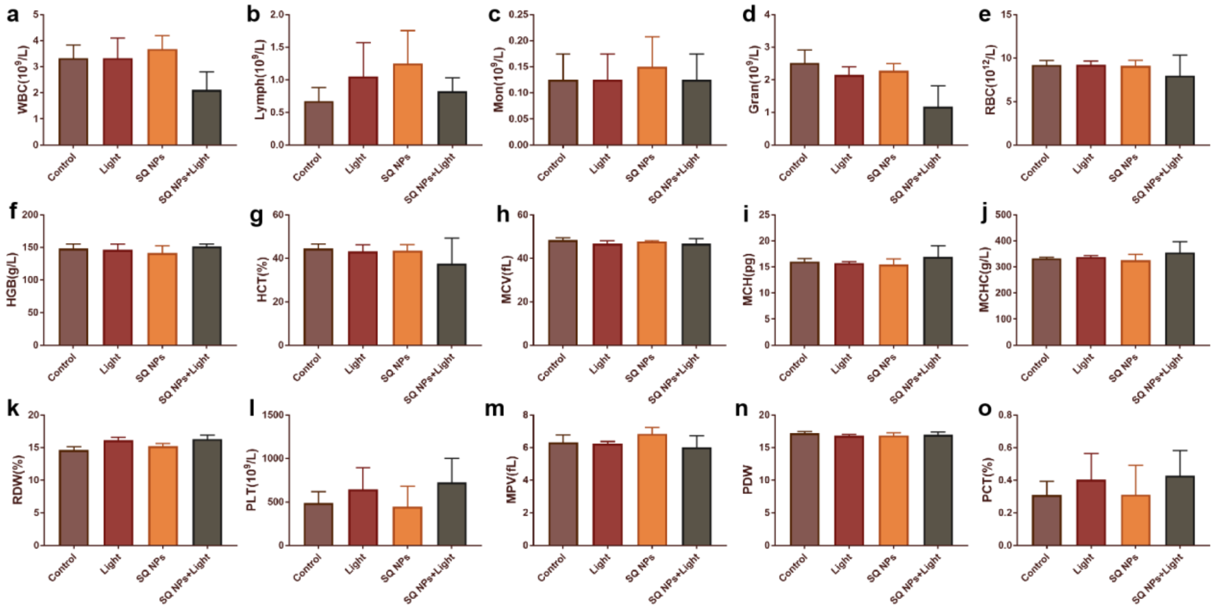

Fig. S11: Hematological analysis for mice in different groups 16 days post-therapy. Data represent means ± SD from three independent replicates. SD is denoted by the error bars.

References

[1] Q. Liu, Z. Xie, M. Qiu, I. Shim, Y. Yang, S. Xie, et al. "Prodrug-Loaded Zirconium Carbide Nanosheets as a Novel Biophotonic Nanoplatform for Effective Treatment of Cancer" Adv Sci. vol. 7, no. 24, pp. 2001191, 2020. https://doi.org/10.1002/advs.202001191

[2] M. Wang, J. Zhu, Y. Zi, Z.-G. Wu, H. Hu, Z. Xie, et al. "Functional Two-Dimensional Black Phosphorus Nanostructures Towards Next-Generation Devices" J Mater Chem A. vol. 9, no. 21, pp. 12433-12353, 2021. https://doi.org/10.1039/d1ta02027g.

[3] S. Liu, J. Pan, J. Liu, Y. Ma, F. Qiu, L. Mei, et al. "Dynamically PEGylated and Borate-Coordination-Polymer-Coated Polydopamine Nanoparticles for Synergetic Tumor-Targeted, Chemo-Photothermal Combination Therapy." Small, vol. 14, no. 13, 2018. https://doi.org/10.1002/smll.201703968.
